# Amaurosis Post-Bilateral Neck Dissection: A Systematic Review

**DOI:** 10.1007/s12070-024-04972-6

**Published:** 2024-08-17

**Authors:** Jafar Hayat, Yahya Ali, Salman Hussain, Mohammad Ramadhan, Maha Al-Gilani

**Affiliations:** 1 Department of Otolaryngology, Head and Neck Surgery, Jaber Al-Ahmad Al-Sabah Hospital, Kuwait, Kuwait; 2 The University of Manchester, Royal Preston Hospital, Preston, UK; 3 Department of Otolaryngology-Head and Neck Surgery, University of Ottawa, Ottawa, ON Canada; 4 Department of General surgery, Jaber Al-Ahmad Al-Sabah hospital, Kuwait, Kuwait

**Keywords:** Amaurosis, Blindness, Bilateral neck dissection, Radical neck dissection

## Abstract

To discuss the prevalence of amaurosis post-bilateral radical neck dissections; and to provide a management algorithm that highlights the approaches undertaken in available literature to minimize incidence and maximally improve outcomes. This objective will be achieved by systematically reviewing and highlighting current literature. We systematically reviewed Pubmed, EMBASE, and Web of Science for articles pertaining to the management of amaurosis post-bilateral neck dissection. A comprehensive search of available literature was conducted by two independent authors to yield 23 articles to be included in the review. Due to the heterogeneity of study designs and outcome measures, findings were summarized and analyzed descriptively. 23 cases were reported between 1960 and 2021; *n* = 21 were reported through case reports. Neck dissection type alongside pharyngectomy (*n* = 7) and laryngectomy (*n* = 13) status were documented. Mean symptomatic onset was 7.3 ± 5.561 days; median onset *n* = 3. Seven reported symptoms post-operative day (POD) 0. *n* = 17 patients underwent bilateral radical neck dissections. *n* = 16 patients reported intraoperative hypotension. Investigations the patients underwent were documented. The most common causes of the disease included posterior ischaemic optic neuropathy (PION) (*n* = 8) and anterior ischaemic optic neuropathy (AION) (*n* = 5). The mean transfused amount of blood was 750 ml. The most common management of amaurosis post-bilateral neck dissection were through high dose corticosteroids. Mannitol and acetazolamide were also documented managements of the condition. Overall, there is a paucity of evidence pertaining to the management of amaurosis post-bilateral neck dissection; highlighting the importance of reviewing the available literature and proposing a management algorithm.

## Introduction

Amaurosis, or partial to total blindness, is a potentially devastating postoperative complication that may occur after bilateral neck dissections. Loss of vision can be identified either immediately postoperatively or over a span of days, weeks, or even months after completion of the surgery. Treating this condition can be optimized with appropriate clinical examinations, laboratory investigations, and management approaches.

Amaurosis occurring post-bilateral neck dissection is an entity that was first described by GA Milner in 1960 [1]. Since the first case report, there have been less than 25 reports created that discuss the complication. Despite the mechanism of injury not being completely understood, explanations as to why perioperative amaurosis occurs have been put forth. The most prominent theory is that of anterior ischemic neuropathy and posterior ischemic neuropathy being the primary causes of this complication [2]; with ischemic neuropathy believed to be largely due to hypotension, anemia and oedema in the venous system [3, 4].

Due to the rarity and the lack of available literature regarding the topic of amaurosis post-bilateral neck dissections, we intend to provide a thorough documentation of the condition by systematically reviewing available literature and discussing the process of diagnosing, investigating, and managing the condition; alongside proposing a management algorithm to both decrease the occurrence of the condition and to optimize management.

## Methods

**Fig. 1:**
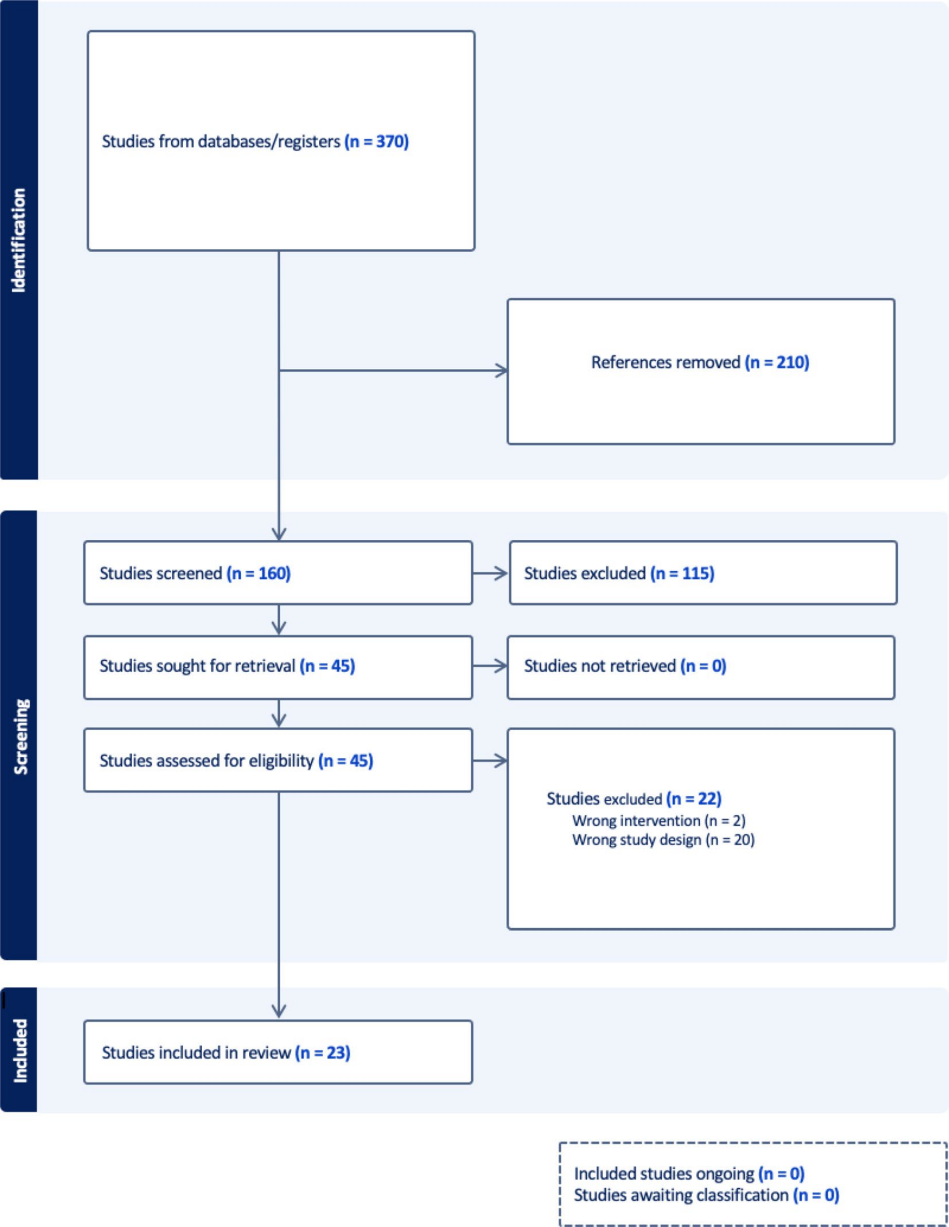
Description of the search strategy according to the PRISMA protocol. This flowchart highlights the search strategy the team underwent throughout their systematic search of available literature

A systematic review of the available literature was conducted under the 2020 PRISMA guidelines. A comprehensive search was conducted by 2 independent reviewers (J.H, Y.A) on three databases: Pubmed, EMBASE, and Web of Science. Discrepancies in screening were resolved through discussions between the two screening authors. Figure 1 depicts the PRISMA protocol relevant to our search.

### Inclusion/exclusion Criteria

*Inclusion criteria* involved all levels of peer-reviewed scientific evidence regarding the topic of amaurosis or vision loss post-bilateral neck dissections in human studies of both sexes and in any age group.

*Exclusion criteria* involved articles with cohorts who had not suffered any form of vision loss after being managed via bilateral neck dissections or cohorts who had undergone unilateral neck dissections regardless of if they had any suffered loss of vision. Other articles that were excluded were articles available exclusively in abstract form only.

### Intervention

Patients had to have undergone bilateral neck dissections as an intervention for any underlying condition.

### Comparator/control

No comparator or control group was required for inclusion in this review.

### Primary Outcomes

Studies were required to report post-operative clinical presentations alongside the types of neck dissections done, and the time in which the amaurosis occurred postoperatively. Ideally, the studies provided information on how they managed the patient once the symptoms began.

### Secondary Outcomes

We also extracted data on the duration it would take for the amaurosis to occur, alongside perioperative blood pressure readings. Status of anemia, whether packed red blood cells (PRBCs) were given at any point, and any other clinical or radiological findings were also extracted and quantitatively and descriptively documented in Table 1.

### Statistical Analysis

Highly heterogeneous results were expected secondary to variations in study design, reporting, suspected diagnoses, managements, and outcome measures. Data pertaining to examination findings and treatment responses were extracted where applicable. If a specific scale or scoring sheet was utilized, then the values were extracted and summarized in Table 1. We were unable to conduct a meta-analysis due to the heterogeneity of the included studies in terms of study designs, protocols, and treatments.

### Risk of Bias Assessment

Studies of all different types (case reports, case series, cohort, case-control and interventional studies) were included. For this reason, no specific quality assessment tool was used. Data that was ambiguous or that did not satisfy the inclusion criteria were excluded from the manuscript.

## Results

A total of 23 patients were reported. The earliest report of amaurosis post-bilateral neck dissection was in 1960, while the latest was reported in 2021. 21 of the patients were reported as case reports whilst two patients were part of a case series. In terms of patient’s characteristics, 13 patients were also reported to have undergone laryngectomies and 7 were reported to have undergone pharyngectomies with their bilateral neck dissections. 63% of the patients (*n* = 17) underwent bilateral radical neck dissections. 9% of the patients (*n* = 2) underwent bilateral selective neck dissections and 9% of the patients (*n* = 2) underwent unilateral radical neck dissections and contralateral modified radical neck dissections. One patient underwent a lateral neck dissection and a modified type 3 radical neck dissection, and one patient underwent a bilateral modified radical neck dissection as depicted in Table 1.

30% of the patients (*n* = 7) had developed amaurosis the same day the surgery was done. Duration to symptomatic occurrence ranged between day 0 to day 60 postoperatively. The mean duration in which amaurosis occurred was 7.3 ± 5.561 days; with the median being 3 days. Intraoperative hypotension was described in 70% (*n* = 16) of the patients. In the reported postoperative blood pressures, 4 studies reported episodes of postoperative hypotension during recovery. 57% (*n* = 13) patients in the study were reported to be anemic.

19 studies conducted ophthalmic examinations on patients once symptoms occurred. 11 of them utilized CT (computed tomography) scans, 6 used MRI (magnetic resonance imaging) scans and 4 used angiography. In terms of blood tests, 3 measured Hb, 3 measured patient ESR and 2 measured hematocrits. 2 studies conducted neurological examinations.

The suspected causes of the amaurosis differed in each study. Most stated posterior ischemic optic neuropathy as the main cause (*n* = 8 studies). Moreover, 5 studies reported anterior ischemic optic neuropathy and 4 reported ischemic optic neuropathy (unspecified) as a cause of patient vision loss. 2 studies reported a hemorrhage and 1 study each reported decreased intracranial blood flow and increased intracranial pressure, occlusion of the central retinal arteries, severe facial oedema causing occlusion and optic nerve atrophy (unspecified) respectively.

Of the patients, 17 received PRBCs. Standardizing the rate of a unit of blood to 250 ml, the average reported blood given to patients requiring transfusion was 1300 ml, or 5.2 units each, while the mean was 750 ml, or 3 units.

**Table 1:** Table highlighting available patient reports of amaurosis post-bilateral neck dissection

Study ID	Type of surgery	Time of occurrence	Preoperative BP of patient (mmHg)	Intraoperative BP of Patient (mmHg)	Postoperative BP of Patient (mmHg)	Anemia status (Yes/No)	What was the suspected cause?	Investigations	Treatment and prognosis	Were there PRBCs given?	Quantity of PRBCs given (Liters)
Milner [1]	Bilateral radical neck dissection	POD 6	135/80	N/A	Hypotension postoperatively (80/60) which then rose to 100/80 after being given blood	N/A	In summary, several factors may have been to blame in this patient, separately or in combination of: 1. Increased intracranial pressure 2. Decreased intracranial blood flow	2 Ophthalmic reviews were done: 1: Vessels show no abnormality. Intraocular tension feels normal. 2nd - Vision nil. Fundoscopy: The picture is that of irreversible optic atrophy.	No management was suggested, patient was permanently blind.	Yes	1.7 intraoperatively, 1.1 postoperatively (2.8 total)
Torti et al. [15]	Bilateral radical neck dissection and removal of the pharynx and laynx	Simultaneously	110/70	BP varied from 120/60 to 75/30.	N/A	No	Bilateral occlusion of the central retinal arteries.	Ophthalmic evaluation and fundoscopy: Chemosis of the conjunctiva with limitation of the ocular excursions. Both pupils were equally dilated, fixed, and without reaction to light. The fundi showed narrowed arteries and veins, pale retinae, and cherry-red appearance of the maculae. Neurological examination - normal	Retrobulbar injection of lidocaine (Xylocaine) 2% was given and repeated three hours later. Vision was totally lost, and remained unchanged.	No	N/A
Chutkow et al. [9]	Bilateral radical neck dissection and total laryngectomy	Simultaneously	N/A	Intraoperative hypotension	N/A	Yes	Amaurosis following hemorrhage.	N/A	N/A	Yes	5.5
Balm et al. [5]	Bilateral radical neck dissection and total laryngectomy	POD 1	N/A	Stable throughout the surgery	N/A	No	Severe facial oedema postoperatively.	Ophthalmic evaluation: Absence of light perception bilaterally and dilated pupils unresponsive to light. Eye movements were unrestricted. Fundoscopy revealed slight papilloedema in the right eye and a normal optic nerve head on the left. No signs of central retinal artery occlusion were seen. Neurological examination: Unremarkable CT scan: There were no radiographic abnormalities of the brain (Fig. 1), but the neuroradiologist noted distended superior orbital veins and dilated veins around the optic nerve and optic apex Hb − 8.8 mmol/L LP - Pressure of 55 cm	The patient remained permanently blind despite our conservative treatment measures	No	N/A
Marks et al. [3]	Bilateral radical neck dissection and removal of the pharynx and larynx	POD 3	N/A	Stable at 140/90 systolic	Profound hypotension over 4 h, ranging from 50 to 80 systolic	N/A	Bilateral intraorbital optic nerve hemorrhagic infarctions. The posterior intraorbital segment completely infarcted and the anterior intraorbital segment partially involved.	Ophthalmic evaluation: 3-mm pupils nonreactive to light with a normal funduscopic examination. Neurological examination and fundus examination - Unremarkable Postmortem examination - revealed a fresh cerebral infarct, not involving the occipital lobe, and postoperative changes.	No immediate management suggested, patient died few weeks later.	Yes	3.15
Kirkali et al. [18]	Bilateral radical neck dissection	1 month post-operatively	N/A	Intraoperative hypotension	N/A	Yes	Posterior Ischemic optic neuropathy	N/A	N/A	Yes	1
Wilson et al. [6]	Bilateral radical neck dissection and total glossectomy and total laryngectomy	POD 0 (6 h post-operatively)	N/A	here were no prolonged periods of hypertension or hypotension	N/A	N/A	Anterior Ischemic optic neuropathy	Ophthalmic evaluation: No light perception and absent pupillary response bilaterally. Applanation tonometry was 25 and 26 mmHg in the left and right eyes, respectively. Fundoscopy: The optic nerves were pale and edematous and the retinal artery and vein were patent.	Methylprednisolone, 250 mg, mannitol, 20 g, acetazolamide, 250 mg, were immediately given intravenously every 6 h and timolol, 0.5%, was administered topically twice a day. Bilateral lateral canthotomies and inferior cantholysis were performed. Despite early and aggressive treatment, the patient’s bilateral complete blindness was permanent.	Yes	1.4
Dodd [19]	Bilateral radical neck dissection	Simultaneously	N/A	Hypotension	N/A	No	Posterior Ischemic optic neuropathy	N/A	N/A	Yes	0.7
Bouzaiene et al. [10]	Bilateral radical neck dissection and composite resection	Simultaneously	N/A	Hypotension	N/A	N/A	PION bilateral	N/A	N/A	Yes	0.7
Schobel et al. [16]	Bilateral radical neck dissection and composite resection	2 months post-operatively (ICU admission and coma post-operatively).	N/A	N/A	N/A	N/A	Optic nerve atrophy (Unexplained)	Fundoscopic findings: Nerve atrophy in both eyes. There was no visually evoked response in either eye. CT, MRI, Selective angiography: All unremarkable	The patient was discharged from hospital with total loss of vision in both eyes.Half a year later, visual acuity had partially recovered in the left eye, but the patient mentioned a tubular restriction of the visual field and he could see only phantoms. No mention of inventions to improve vision.	N/A	N/A
Le Queau [11]	Bilateral radical neck dissection and buccal tumorectomy	POD 1	N/A	2 episodes of hypotension (70 for 5 min and 80 for 5 min).	N/A	Yes	Bilateral Posterior ischemic optic neuropathy	Ophthalmic examination: Bilateral mydriasis with absence of photomotor reflex, without associated neurological signs elsewhere. The eye did not show any signs of occlusion of the central arteries of the retina, nor any sign of papilledema. Brain CT: Bilateral exophthalmos with moderate retrobulbar oedema. Brain MRI: No sign of cerebral ischemia or decompression of the optical pathways along their entire path.	Despite treatment with high-dose corticosteroids and vascular antispasmodics, blindness remained complete and definitive,	Yes	0.7
Strome et al. [20]	Bilateral modified radical neck dissection	POD 2	N/A	Several episodes of hypotension with the lowest being 87/45	N/A	Yes	Anterior Ischemic optic neuropathy	Ophthalmic examination: Hand-motion vision in the right eye and 20/30 vision in the left eye. He had a left relative afferent pupillary defect, constricted left visual field, and a swollen left optic nerve head.	No further treatment was recommended, because the patient was transfused, and blood pressure was normalized. The patient’s vision remained stable for 1 week, then gradually improved. At 6 weeks post- operation, the patient’s visual acuity in the left eye returned to its 20/20 preoperative state. His hand motion vision in the right eye was un- changed and attributed to cataract.	Yes	0.35
Pazos et al. [4]	Bilateral radical neck dissection, total laryngectomy, partial pharyngectomy	POD 20	130/80	Hypotension for 30 min (systolic around the low 80s)	N/A	Yes	Posterior ischemic optic neuropathy	Ophthalmic examination: No light perception and a failure to react to visual threats bilaterally. The pupils were dilated bilaterally to 7 mm and were nonreactive. Extraocular movements were intact. The anterior segment appeared normal except for the presence of resolving chemosis. Intraocular pressures were 15 in the right eye and 13 in the left. The posterior segment examination showed normal-appearing maculae and optic nerve heads, well-perfused arterioles, and normal-diameter venules. No retinal hemorrhage or edema was seen in either eye. Visual evoked potential: Unremarkable CT scan: Unilateral hypodensity of the occipital lobe, ventriculomegaly, and generalized atrophy.	No treatment proposed. Vision remained no light perception in both eyes.	Yes	2.8
Go ¨tte et al. [7]	Bilateral radical neck dissection and a total laryngectomy	POD 5	N/A	Uneventful; no episodes of hyper/hypotension	N/A	Yes	Bilateral Anterior ischemic optic neuropathy	A duplex sonogram of both carotid arteries, an MRI, and a magnetic resonance angiogram all showed unaltered perfusion of all major feeding vessels to the brain. CT scan: No signs of infarction or metastases. Ophthalmic examination: Pallid, edematous discs bilaterally on fundoscopy. Extensive visual field loss was found on perimetric examination. Visual acuity was hand movements in the right eye and 20/200 in the left eye. Intraocular pressure: normal. ESR slightly elevated (25 mm)	The patient received low- molecular-weight heparin (nadro- parin calcium [Fraxiparine]) during the entire postoperative period. He also received 40 mg of cortisone acetate (Fortecortin) intravenously after his visual loss developed. However, the patient continued to have only hand motion vision in both eyes.	N/A	N/A
Mamede et al., [12]	Lateral neck dissection on the left, modified type 3 radical neck dissection on the right and a total laryngectomy	POD 5	130/70	100/60, had episodes of hypotension (80/45)	N/A	Yes	Bilateral anterior ischemic optic neuritis due to alteration in cerebrospinal fluid pressure due to head positioning	Ophthalmic evaluation: Reduced intrinsic reflexes, with cloudy papillae and edema of the optic disk. Hemoglobin: 10.3 Haematocrit: 32.6%	No management proposed. No visual improvement occurred over the subsequent days.	Yes	0.3
Fenton et al. [21]	Radical neck dissection on the right, modified radical neck dissection on the left with preservation of the internal jugular vein	Simultaneously	N/A	3 episodes of hypotension with the lowest being 80/40	N/A	Yes	Ischaemic optic neuropathy	Ophthalmic examination and fundoscopy: No perception of light in the right eye and 6/6 vision in the left eye. He had a relative afferent pupil defect in the right eye. Intraocular pressure: Normal Posterior fossa CT - Unremarkable	No management proposed. At follow-up six weeks later, there was no perception of light in the right eye.	Yes	2.45
Fenton et al. [21]	Bilateral selective neck dissection with rim mandibulectomy and excision of the floor of the mouth. Reconstruction was with a radial forearm free flap	POD 7	N/A	3 episodes of hypotension for about 5 min, one at 80/45 and 2 at 80/60	N/A	No	Ischaemic optic neuropathy	Ophthalmic examination and fundoscopy: 6/9 vision in each eye. Visual Fields to confrontation revealed a superotemporal field defect in the left eye and a full visual field in the right eye. Pupillary reaction in each eye was normal and fundoscopy was unremarkable. MRI: Unremarkable. Goldman field analysis: Superotemporal altitudinal field defect	No management proposed. Follow-up two months later showed no change in either the visual acuity or the visual eld defect of the left eye.	Yes	0.7
Worrell et al. [2]	Bilateral radical neck dissection and a total laryngectomy	Simultaneously	N/A	2 episodes of severe hypotension (unquantified). Otherwise, BP was around 80–100 systolic throughout	N/A	Yes	Posterior ischemic optic neuropathy	Ophthalmic examination and fundoscopy: The absence of light perception was confirmed. Funduscopic examination was normal. Pupillary reflexes were absent, and extraocular movement was limited. The patient’s intraocular pressures were at the high end of normal, and so it was elected to perform bilateral lateral canthotomies. CT scan: Normal	bilateral lateral canthotomies for high intraocular pressure. The patient’s facial and pulmonary edema improved over the next few weeks, so he was transferred to a basic level of care safely and commenced postoperative radiotherapy. No mention of treatment for visual improvement or prognosis of vision.	Yes	0.35
Obuchowska et al. [13]	Bilateral radical neck dissection and a total laryngectomy	POD 5	N/A	One hypotensive episode (65/30)	N/A	Yes	Posterior ischemic optic neuropathy	Ophthalmic examination and fundoscopy: Visual acuity in the right eye about 4/50, in the eye left; counting fingers from 2 m, impaired colour vision in both eyes, impaired reaction of both pupils to light and a normal fundus.	Due to the time that has passed since then onset of symptoms, and no documented effectiveness methods of treating this neuropathy were left to the patient without ophthalmological treatment, recommending only periodic examinations control.	Yes	1
Suárez-Fernández et al. [17]	Bilateral radical neck dissection and a total laryngectomy	POD 2	100/70	75/50 to 100/75 range during surgery (hypotensive)	115/55	Yes	Anterior ischemic optic neuropathy	Ophthalmic examination: Hand movement in the RE and 20/50 in the LE. The pupils were equal in size, but a relative afferent defect was found in the RE. Fundoscopy: A swollen optic disc with small hemorrhages in the RE and a swollen optic disc in the LE. Sedimentation rate: 113 mm (20–40 mm) Protein C: 16.97 mg/dL (0.5–2 mg/dL), Hb: 9.2 g/dL Hematocrit: 26.6% CT scan: Unremarkable	The patient was treated with methylprednisolone 1 g for three days, followed by 1 mg/kg/day for 10 days. VA improved the second day of treatment, rising to 20/200 in the RE and 15/25 in the LE, but on the follow- ing days it decreased again to hand movements in the RE.	No	N/A
Crockett et al. [8]	Bilateral radical neck dissection and a total laryngectomy	POD 14 (was operated on again for a bleeding IJV).	N/A	N/A	N/A	N/A	Non-arteritic ischaemic optic neuritis secondary to acute hypoxia caused by substantial blood loss as a result of his jugulo-neopharyngeal fistula.	Ophthalmic examination: Reduced visual acuity bilaterally (right eye acuity = 6/5; left eye acuity = 6/12) and reduced left eye color vision. There was a left-sided relative afferent pupillary defect with bilateral peripheral visual loss. CT scan: Unremarkable	The patient was treated with oral corticosteroids for 10 days. Follow up at six months revealed no further improvement in the patient’s vision.	N/A	N/A
Krasinkova et al. [14]	Unilateral radical neck dissection and a contralateral modified radical neck dissection and a hemimandibulectomy and a partial pharyngectomy	POD 4	140/80	Two 20-minute hypotensive episodes where systolic was 70 at its lowest	Unspecified instability in BP for 45 min	Yes	Ischemia of the optic nerve due to intraoperative hypotension	Ophthalmic examination: Chemosis in the anterior segment and a swelling of the head of the optic nerves of both sides in the posterior segment. CT scan: Unremarkable MRI scan: A scattered infarction in the supply area of the medial cerebral artery of the left side was discovered. MR Angiography: Unremarkable	A high-dose intravenous cortisone treatment of 1000 mg for three days was started on the fifth postoperative day but vision did not recover	Yes	0.7
Kohyama et al. [22]	Bilateral selective neck dissections (levels 2–4) with preservation of the IJV bilaterally alongside a laryngectomy and pharyngectomy	POD 3	150 systolic	> 100 systolic but had unspecified hypotensive episodes throughout	100 systolic but had an episode where it dropped to 60	Yes	Perfusion to the optic nerve compromised by increased ICP due to compression of the internal jugular vein bilaterally (Posterior ischemic optic neuropathy).	Ophthalmic review: Lack of afferent pupillary light reflex and visual acuity of light perception only. Fundoscopy: Unremarkable. MRI: Unremarkable. MR Angiography: Unremarkable.	1 mg/Kg/day Prednisolone for 2 months +, no improvement; vision level remained at light perception.	Yes	N/A

This table contains all the studies identified in our systematic review and contains relevant information regarding type of intervention employed, investigations ordered, and measurement outcomes used in the study

## Discussion

Amaurosis is a complication that requires urgent management to mitigate progression. Following the reported articles available in literature, most of the patients included in this study had common experiences.

Whilst most patients reported at least one episode of hypotension either before, during or after the surgery, only 3 studies mentioned blood pressure being stable throughout the patient’s hospital stay [5–7]. Several patients were reported to have taken longer than usual for a bilateral neck dissection [1–17]; with the longest taking 23 h from start to finish [4]. More than half of the patients were anemic (< 7.0 g/dL of hemoglobin) and a significant number of patients were reported to have high intraoperative and/or postoperative blood loss, hypotension, or both. To manage the anemia, hypotension, and significant blood loss, many were managed with packed red blood cells (PRBCs) [1–4, 6, 9–14, 18–23].

In most instances, patients developed amaurosis within a week of the operation, including 6 patients who reported blindness immediately after the procedure [2, 9, 10, 15, 19, 21].

Many explanations as to the cause of blindness have been put forth with the overwhelming majority of patients suggesting hypotension having a significant role in the deterioration of the patients [1–4, 8–22]. Ischemic optic neuropathy is thought to be a primary mechanism for blindness in patients undergoing bilateral neck dissections. The underlying cause for the condition varied depending on the study. Anemic status of the patient, hypotension, and increased congestion in the venous system were all linked to blindness [3, 4].

The two main types of ischemic optic neuropathy are posterior ischemic optic neuropathy (PION) and anterior ischemic optic neuropathy (AION). According to Kohyama et al., it is thought that the mechanism of injury in PION is multifactorial that includes hypotensive episodes, anemia, lengthy procedure times, and administering large volumes of fluid; alongside pressure due to the ligation of the internal jugular vein [22].

The first case of AION was documented by Wilson et al. in 1991. In this case, there was immediate AION following the surgery. This was believed to be secondary to hypertension and the use of bubble-type eye protection [6]. This was the only case to report hypertension as the cause of AION as opposed to hypotension. Götte et al. suggested that AION is more likely to occur after multiple hemorrhages and interruptions in blood flow rather than one substantial event [20].

Investigations to identify the source of the amaurosis were documented in all studies. In most patients, the first investigative measure involved an urgent ophthalmological review of the patient. Conjunctival swelling and unresponsive, dilated pupils, and an overall reduced visual acuity were reported in most patients [1–8, 11–17, 20–22]. Wilson et al. reported that their patient with AION had a pale and edematous optic disc and a significant increase in intraocular pressure [6]. In PION, dilated and tortuous retinal veins with slightly elevated intraocular pressures were identified [4]. In certain instances, brain imaging was utilized to exclude any neurological/brain pathology. The modality of scans differed between studies; but in most, CT or MRI scans were utilized. Although most CT scans performed were unremarkable, one study documented exophthalmos with retrobulbar oedema, and another documented a unilateral hypodensity in the occipital lobe and ventriculomegaly; both scans were on patients diagnosed with PION [4, 11]. All MRI scans performed were unremarkable except for one case which found an infarction in the supply area of the left middle cerebral artery. This led to the diagnosis of ischemia of the optic nerve due to intraoperative hypotension [14]. In certain instances, a full neurological examination was performed but all examinations documented unremarkable findings [3, 5, 15].

In managing the amaurosis, very little was documented for patients to improve visual acuity in older reports; however, in newer studies, most patients were given varying doses of steroids either orally or intravenously with mixed outcomes reported. While several patients reported symptomatic improvement others reported no changes in visual acuity post-administration of the steroid. One study by Suárez-Fernández et al. suggested some improvement in visual acuity on the second day of treatment. Visual acuity increased to 20/200 in the right eye and 15/25 in the left eye, only to deteriorate again to hand movements in the right eye in the following days [6, 8, 11, 14, 17, 22].

In a case where one patient experienced elevated intraocular pressure, a canthotomy was performed to relieve pressure [2].

Although the data showed mixed results, the use of steroids may prove most beneficial to patients’ visions; with Kohyama et al. utilizing a dose of 1 mg/kg/day of prednisolone for 2 months and Suárez-Fernández et al. utilizing a dose of 1 mg/kg/day of methylprednisolone for 10 days [17, 22]; and Karsinkova et al. utilizing a high-dose corticosteroid (1000 mg) for 3 days [14]. For patients who experience elevated intraocular pressure, mannitol and acetazolamide can be of use [6].

**Fig. 2:**
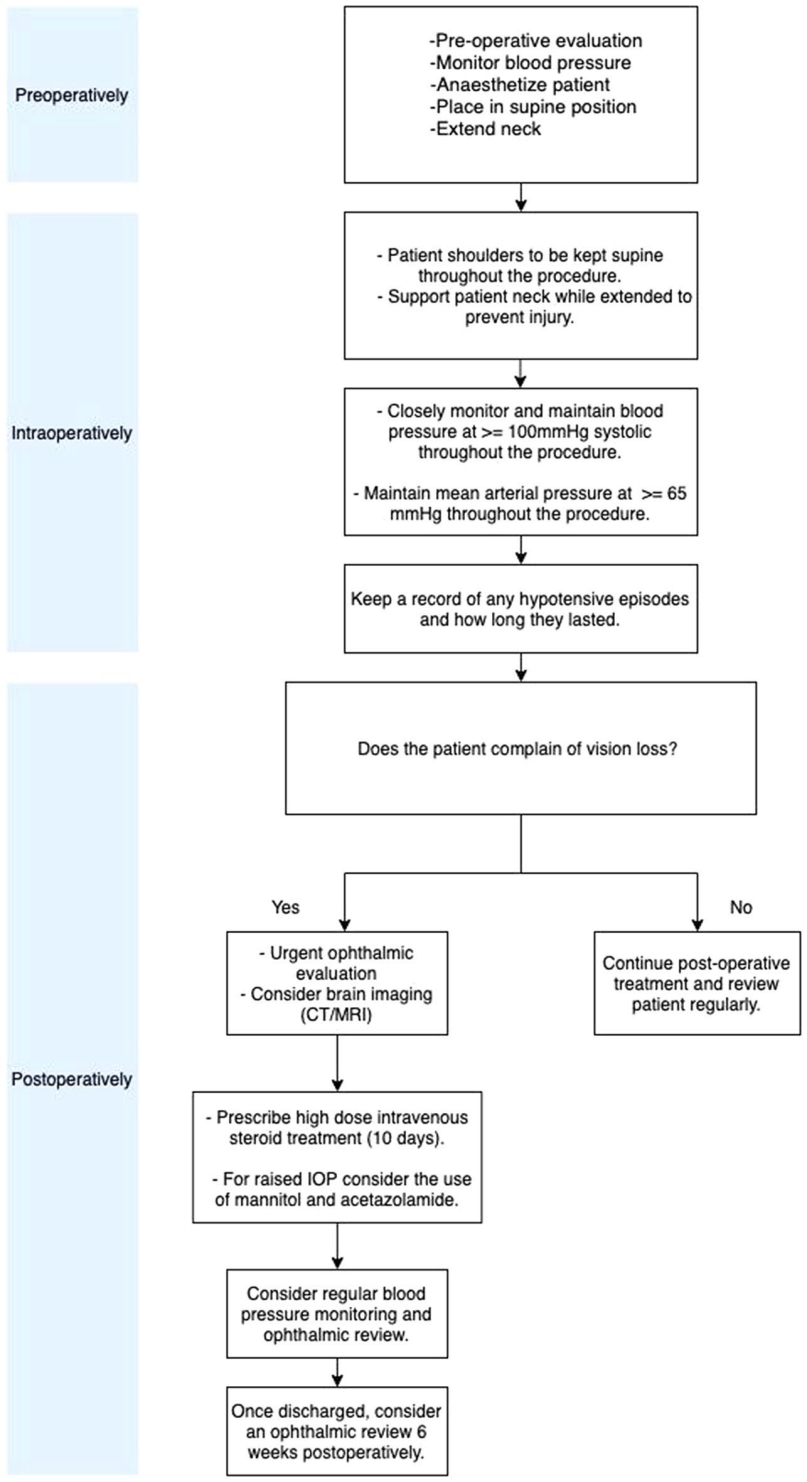
Proposed algorithm in mitigating and managing amaurosis. This algorithm is a proposed algorithm for clinicians to consider in minimizing the incidence of amaurosis fugax for patients undergoing bilateral neck dissections

Due to the importance of preserving patient eye functions, we have proposed a management algorithm depicted in Fig. 2. While in the overwhelming majority of patient reports the amaurosis was irreversible; taking appropriate perioperative measures and quickly managing the condition are among the most commonly employed methods to successfully mitigate complications.

The limited sample size makes it difficult to highlight trends in the outcomes of patients based on the intervention used. The available data demonstrates, however, that with the right perioperative approach and management, clinicians will be able to mitigate adverse outcomes and improve patient care.

## Conclusion

Amaurosis is a rare albeit crippling postoperative complication of bilateral neck dissections, with less than 25 reports being available in literature. Due to the severity and nature of the complication, clinicians should document it as a potential risk of the surgery when consulting patients.

The most common method of managing amaurosis post-bilateral neck dissection were through high dose corticosteroids. Mannitol and acetazolamide were also documented managements of the condition that may be utilized on a case-by-case basis.

Overall, there is a paucity of evidence pertaining to the management of amaurosis post-bilateral neck dissection, highlighting the importance of reviewing the available literature and providing guidance on the management of the condition.
